# Student Poster Conferences as a Formative, Authentic, Inclusive and Sustainable Assessment Practice

**DOI:** 10.1111/tct.70050

**Published:** 2025-03-03

**Authors:** Danica Sims, Catherine Swales, Debbie Aitken

**Affiliations:** ^1^ Department of Education University of Oxford Oxford UK; ^2^ Biomedical Engineering and Healthcare Technology Research Centre, Faculty of Health Sciences University of Johannesburg Johannesburg South Africa; ^3^ Nuffield Department of Orthopaedics, Rheumatology and Musculoskeletal Sciences University of Oxford Oxford UK

**Keywords:** assessment, authentic assessment, community of practice, formative assessment, inclusive assessment, poster conference, sustainable assessment

## Abstract

Innovative assessment for learning is an important part of professional medical and health professions education programmes. We propose that student poster conferences are a formative, authentic, inclusive and sustainable assessment practice that has the potential to support research competency development and professional identity formation. Of primary importance, the poster conference is a safe‐to‐fail formative experiential learning opportunity, with opportunities for feedback from peers and key stakeholders. It is a form of authentic assessment that replicates a ‘real world’ conference experience and aspects of public scholarship and peer review. Inclusivity is possible through the flexibility, diversity and potential multimodality of the poster and presentation formats. Furthermore, by opening up the event to other students, staff and health professionals, it is inclusive, while simultaneously including students into the larger Communities of Practice through legitimate peripheral participation. Lastly, it is a sustainable form of assessment as it equips students for future research practices and dissemination. As a relatively low‐cost assessment practice, compatible with online formats too, poster conferences are a widely applicable assessment method across contexts, disciplines and study levels.

## 1 What is the Assessment Challenge?

When it comes to assessment in medical and health professions education, one of the key goals of educators is to use their assessment to drive student learning and development in desirable ways.

In 2021, the University of Oxford established a new Masters programme in Medical Education. The programme runs over 2 years and is part‐time, with students as full‐time health practitioners, or practitioner–educators, from several healthcare disciplines and contexts. This programme is often their first exposure to medical and health professions education *research*. Extending their research competency into new domains of social sciences and qualitative research can be uncomfortable and stressful for them. We propose that student poster conferences are a *formativ*e, *authentic*, *inclusive* and *sustainable* assessment practice (Box 1).

BOX 1 **Key assessment terms defined**

• **Formative assessment:** Low stakes or safe‐to‐fail (likely not graded) assessments that primarily focus on the learning and feedback opportunity. Formative assessment is also referred to as assessment *for* learning, as opposed to high stakes (decisions, judgements or consequences attached) summative assessment, which is referred to as assessment *of* learning [1, 2, 3, 4, 5].

• **Authentic assessment:** Assessment that replicates relevant and meaningful real world (i.e., ‘authentic’) challenges and evaluates complex, integrative and direct (vs. proxy or indirect) competency. Authentic assessment is forward‐looking and offers value beyond the immediate assessment opportunity, as it enables future professional performance, motivating deep engagement [6, 7].

• **Inclusive assessment:** Assessment that is proactively accessible (flexible, multimodal, equitable, nondisadvantaging nor discriminating), allowing for diverse learners to produce or perform in diverse ways [8, 9]. Additionally, assessment that is welcoming and encourages participation and belonging to a larger community.

• **Sustainable assessment:** Assessment that contributes to learning beyond the immediate assessment, course or time frame, preparing students to meet future learning needs (i.e., doing a ‘double duty’ of meeting both present and future learning needs, where learners demonstrate ‘real’ competency [referring to research related knowledge, skills and attitudes] and develop as lifelong learners) [1, 10].

## 2 What is the Proposed Solution?

In the second year of the programme, students are asked to develop and present their research (or research in progress) as a poster at our student medical education poster conference. This is based on well‐established practices from other professional programmes within our department.

**FIGURE 1:**
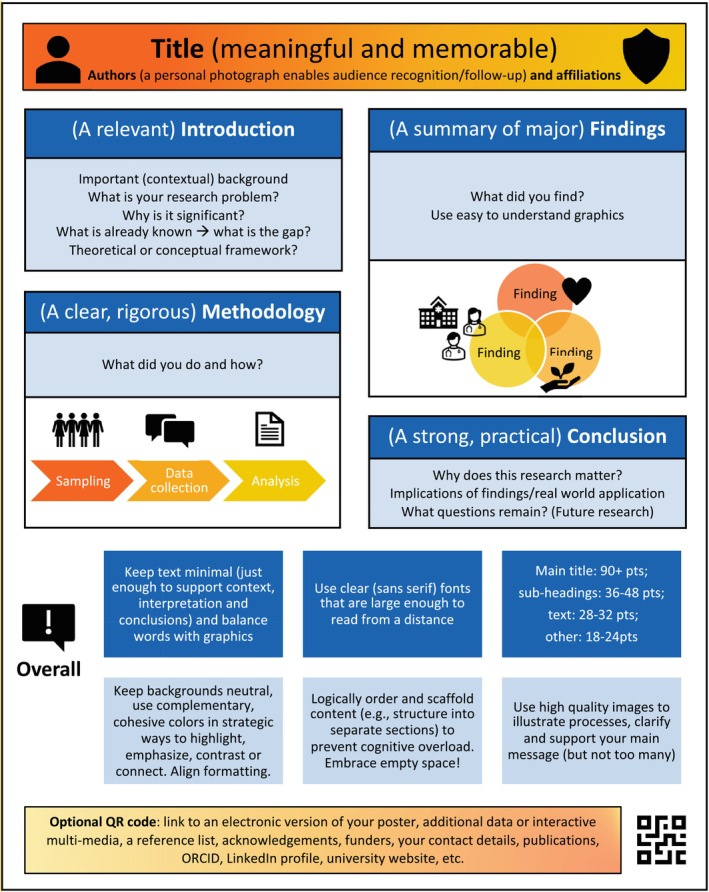
How to design a conference poster [11, 12].

The poster conference is a **formative** assessment opportunity, while participation is compulsory, student posters are not marked for formal grading purposes—although criteria are provided to transparently and explicitly communicate expectations and to scaffold the development of the posters (Figure 1, Box 2) [1, 7]. It is meant to be a low‐stakes and safe‐to‐fail learning opportunity. For example, if the research projects are incomplete (i.e., in progress) or if the nerves get the better of the presenter, there remains a point of educational reference that opens avenue for discussion; the presenter is less exposed while retaining the opportunity for stepping into more formal scientific educational discourse and developing those skills.

BOX 2 **Examples of guiding criteria for poster design and presentation**

• **Content, knowledge and expertise:** The poster and presentation demonstrate understanding of the topic, present accurate, in‐depth knowledge, expertly handle questions and provide clear answers.

• **Clarity and organisation:** The poster is a well‐organised ‘visual abstract’ that is easy to follow and clearly communicates the key points. The information is organised in a logical and coherent manner.

• **Visual design and aesthetics, and/or interactive aids:** The poster design is visually appealing and accessible, with appropriate use of text, font size, colours and images (see Figure 1) that are relevant and supportive of the content, enhancing understanding and engaging the viewer.

• **Scientific rigour and quality:** The underlying research project is rigorously designed, and the quality of the work is clearly communicated.

• **Presentation skills:** The presenter engages the audience with a clear, confident, articulate and promotional delivery; maintains a strong presence; and effectively uses body language and voice modulation.

• **Time management:** The presenter stays within the allocated time limit and effectively manages the presentation time, keeps the pace appropriate and ensures all key points are covered.

• **Overall impression and impact:** The poster and presentation make a significant and memorable impact, effectively and persuasively conveying the importance and relevance of the educational research.

It is meant to be a low‐stakes and safe‐to‐fail learning opportunity.

Students are asked to make the most of this learning opportunity (e.g., critically reflecting on how research can be communicated effectively and how to constructively participate within scholarly and knowledge dissemination processes) through the designing of posters, planning of presentations and the giving and receiving of feedback from peers, supervisors and the public (other students, staff and health practitioners). Students are encouraged to use the feedback provided in the development of their summative dissertations. These practices further align with the educational goals of critical evaluation and metacognition development, collaborative learning and stakeholder engagement.

Students are encouraged to use the feedback provided in the development of their summative dissertations.

Requiring students to independently design and present posters in a conference format characterises the activity as **authentic** because their preparations (e.g., engaging in a rich task that requires using a range of knowledge, skills and attitudes, be creative and innovative, self‐evaluating; producing something new that captures their work over several months; publicly presenting work; and answering questions) equip them for ‘real world’ research and scholarship activities [6, 7]. Authentic assessment should encourage deep engagement and learning.

[Authentic assessment] equips them for “real world” research and scholarship activities.

Creation of a poster aligns with the upper levels of Bloom's taxonomy of learning, as students analyse, evaluate and produce a research output. Moreover, designing and presenting a poster in a conference format reaches the upper levels of Miller's amended pyramid of ‘does’ and ‘is’, speaking to the domain of professional identity development [13]. Research competency and the development of a researcher‐identity are programme aims; therefore; the requirement to students to produce such an output is fundamental element of constructively aligned assessment strategy.

**Inclusivity** is seen through the flexible guidance provided and the welcoming of diverse and creative poster designs (Figure 1)—students have the autonomy, freedom and choice to decide on the topic of their health professions education research project, including a range of equity, diversity and inclusion (EDI) topics, and how they want to express their research (i.e., in a varied, personalised and authentic of ways) [8, 14]. Additionally, regardless of research project topic, all students are encouraged to consider EDI issues within their research (i.e., be critically conscious and reflexive).

Students have the autonomy, freedom and choice to decide on the topic of their health professions education research project.

Inclusivity, in terms of accessibility (e.g., physical, financial, audio and/or visual, inclusive design), is additionally possible with the option of a multimodal format (e.g., printed or digital posters, in‐person, online or hybrid events), which strives to increase participation [15]. For example, with digital posters, posters can be provided to attendees *before* the event, interactive elements can be embedded (e.g., images, audio or video clips, alterative text), and zooming in and out of the poster is possible [15].

Opening up the student conference to the public (first year students, academic staff, supervisors, undergraduate medical students, prospective students and health professionals) is inclusive through diversification of their anticipated audience and the receipt of broader feedback from different constituencies and academic communities. We could further argue that it is inclusive towards our students as researchers, as a form of legitimate peripheral participation within the broader community of practice [16].

It is inclusive towards our students as researchers … [through] participation within the broader Community of Practice.

Lastly, the poster conference format is also a **sustainable** assessment method [1, 10] as it prepares students not only for their summative dissertation submission but also, more importantly, for their future, self‐regulated research and scholarship. Moreover, there are benefits beyond the formative poster conference [10] as students can upload their posters to the university's open access research repository (attaching a DOI, ‘digital object identifier’, qualifying it as a legitimate citeable research output) or even reuse their posters for ‘real’ conferences.

## 3 How was the Solution Implemented?

To implement a poster conference, a number of considerations should be kept in mind: What is the purpose of the event, who will be involved (in both organising and participating), what format will it take place, if funding is necessary and so on (Box 3). Depending on these considerations, implementation will vary. For instance, for a solely in‐person event, make sure then venue is large enough for the expected number of students and attendees to safely and comfortably mingle, while ensuring that there are enough boards on which to display the printed posters. In contrast, for a hybrid or online event, digital posters of a high quality should be loaded prior to the event and projected on screens that are large enough for the attendees to see, with the option of smaller printed versions with QR codes to access the digital versions available too [15]. Moreover, an inclusive and participatory approach, actively involving stakeholders, should be adopted when developing a poster conference. In this case, ongoing dialogues and shared decision‐making with students took place to co‐develop and evolve the poster conference. For instance, despite the formative nature of the event, students expressed a desire for there to be ‘awards’ for ‘best poster’ and ‘best presenter’ which we presented at the close of the conference.

BOX 3 **Practical tips for organising a poster conference [15]**

• Be clear on the purpose of the event and if a poster conference is appropriate.

• Put together a (diverse) organising team (i.e., staff and students).

• Choose a suitable platform (e.g., in‐person, online or hybrid).

• Source and organise the necessary resources (e.g., funding, a physical venue, poster display boards, catering, digital screen/s, videoconferencing software, i.e., Microsoft Teams, Zoom, Google Meet and electronic repository to store and access digital posters).

• Provide clear instructions to participants, with all necessary details (e.g., guidelines for poster design and presentation, including accessibility, what information their poster can contain, when to submit or print their posters, when they are presenting, in what format and for how long).

• Advertise the event (e.g., emails, social media and snowballing).

• Manage the poster session (e.g., opening and closing, time keeping and moderation, technical troubleshooting).

• Remember post‐poster conference communications (e.g., feedback from participants and attendees, disseminating knowledge gained with the wider community, accessing and sharing posters after the event).

[For] an inclusive and participatory approach, actively involving stakeholders should be adopted when developing a poster conference.

In terms of instructions provided for participants, students were given practical, but flexible, guidelines and criteria on how to design a poster (Figure 1, Box 2) [11] while also encouraged to be creative (i.e., there were no ‘rules’). Research project topics were grouped into ‘themes’ for the design of a structured conference ‘programme’ (however unstructured programmes are a possibility too). Students were given a total of 5 minutes: 3 minutes to present their poster and 2 minutes for questions from other participants and attendees. We held a hybrid event, with digital posters projected for the oral presentations, as well as printed posters for the ‘poster walk’. After all the students presented, we had an unstructured ‘poster walk’ where attendees were able to revisit printed posters of interest for further informal engagement and discussion. Importantly, first year students from the masters program.

## 4 What Lessons Were Learned That are Relevant to a Wider Global Audience?

In short, poster conferences are a relatively low‐cost, formative, authentic, inclusive and sustainable assessment practice, suitable for students across study levels. However, do not underestimate the organisation and potential resource requirements of such an event (Box 3). Depending on the size of your cohort, a refreshment break can be provided, which affords further opportunities for networking in a less formal setting—which both participants and attendees expressed as worthwhile.

Poster conferences are a relatively low‐cost, formative, authentic, inclusive and sustainable assessment practice.

While there are benefits to solely in‐person and solely online formats; however, based on student preferences and pragmatic challenges (e.g., students unable to travel), we ended up moving from an in‐person to hybrid format. Students printed a physical poster, as important for authentic and experiential learning, and used it during the ‘poster walk’ to facilitate dynamic interactions and rich discussions (e.g., second year students providing practical advice to first year students on issues of ethics, research design, participant recruitment, data collection and analysis), while also providing a digital version for projection on a screen for hybrid presentations.

Do not underestimate the organisation and potential resource requirements of such an event.

**FIGURE 2:**
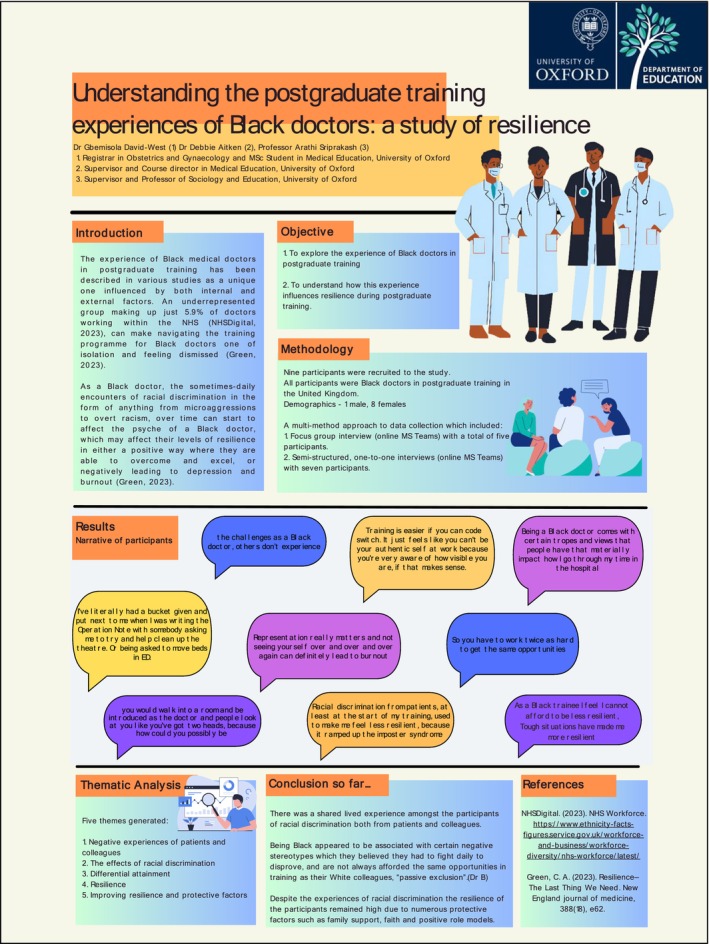
An example of a student poster [17].

In terms of sustainability, students were encouraged to reuse their posters at other medical and health professions education research conferences to build their research confidence and outputs. We also obtained consent from students for use of their digital posters on the university's learning management system/virtual learning environment, for other students to consult when developing their own research ideas and posters. Additionally, students were able to submit their posters to the university's research repository (for DOI assignment), which they appreciated (Figure 2).

## 5 What are the Next Steps?

As this programme continues to evolve, involving students in the design and implementation of this poster conference remains of central importance. While informal feedback has been obtained, through conversations and online forms, formal research and evaluation of this event are planned, for instance, exploring its effect on student learning and development as researchers and their professional identity formation (or change).

While we opened the poster conference up to the wider public, we can improve stakeholder engagement and advertising to encourage greater participation by the public. Lastly, and excitingly, there is potential to build on this programme poster conference into a larger health professions education conference with external participants.

## Disclosure

The authors have nothing to report.

## Data Availability

Data sharing not applicable to this article as no datasets were generated or analysed during the current study.
